# The Institutional Worker

**Published:** 1919-11-22

**Authors:** 


THE
### INSTITUTIONAL WORKER
**Being a Special Supplement to "The Hospital"**

### OUR BUREAU OF INFORMATION.

### Rules for Correspondents.

1. Every letter must be accompanied by the coupon to be cut from the back cover (inside page) of THE HOSPITAL, current issue, and must contain the name and address of the correspondent with pseudonym for publication if desired. Replies by post cannot be given save under exceptional circumstances at the Editor's discretion.

2. Letters from Approved Homes in reply to special needs published in the Bureau should state terms and full particulars, and be sent prepaid under cover to the Editor of the Bureau with name written across coupon for identification.

3. Proprietors of Homes which have not yet been entered on the List of Approved Homes, but have spare accommodation likely to suit special needs, are invited to write for an application form for registration. The fee for registration, which includes two announcements of the Home in the Bureau and other privileges, is 10s.

4. All communications to be addressed to the Editor of THE HOSPITAL, 28 Southampton Street, Strand, London, W.C. 2, and marked "Bureau of Information."

### INSTITUTION FACTS AND FIGURES.

### The Question Box.

#### A CONVALESCENT HOME TIME-TABLE.

THE question was :

**Draw up a daily programme with suitable suggestions regarding exercise, light work, amusements, rest hours and diet to be adopted in a Convalescent Home for working girls situated in the country, and occupied mainly by severe cases of anæmia and debility. The ages of the girls vary from 13 to 30, and as the staff consists of matron and cook-general only, each patient, unless confined to bed, is expected to help with light household tasks. The average number of patients is 12. A plentiful supply of milk and eggs is available.**

E. S. WRITES :

In a home of this description the spirit of "help one another" should be the prevailing feature, and if the example be set by the matron in charge, its influence will soon be subconsciously felt by all its occupants, thus rendering small duties which might otherwise be felt as irksome tasks, into labours of love and service.

Each girl who is well enough to undertake these duties, may be asked to take a weaker girl under her care. In this way she would be giving very valuable assistance to the matron. She could carry up the meals of the friend she undertook to help if she was in bed, dust her room, take her water to wash, and make her bed. The time spent with her friend while doing these small services would help to while away the tedious hours of the more helpless girl. The girls should also take other duties in turn, thus giving them variety and enabling each one to get the plentiful supply of light and outdoor exercise that these cases require.

The day should commence at 7 A.M., when all patients who are well enough to be up should be called. When dressed they may each go to the kitchen for a cup of hot milk, taking a cup to those unable to rise so early. They may then dust the room and lay the dining-table for breakfast, each girl preparing the tray for the sick one under her care. Breakfast should be taken at 8 o'clock, superintended by the matron, and may consist of coffee, milk, bread-and-butter, and eggs, varied some days with bacon if procurable, also marmalade and jam, better still fresh fruit if possible. The washing-up after breakfast may be undertaken by two of the girls, whilst others may prepare vegetables or fruit required for the dinner. The distribution of these duties should be as equal as possible, and care taken that the same girl does not take the same duty for more than two successive days, or it will become monotonous. Each girl will, of course, make her own bed, take her share of keeping the joint bedroom or dormitory neat and clean. A light lunch of milk or cocoa and bread-and-butter may be taken about 11 A.M.; this should be taken out of doors when weather permits, and after this the girls may take walks or rest in the garden until about 1 o'clock, when they should lay the table for dinner at 1.30, and again get trays ready for those still in bed. Dinner should consist of meat or fish and vegetables, with milk pudding and fruit in season; custard may sometimes be served as a change from the usual milk pudding. The after-dinner washing-up is again done by the girls in turn, the afternoon being free for them to spend as they please, either taking walks or resting in the garden with some reading or sewing; some might perhaps like to help in the garden with such work as weeding or tidying up flower-beds. Another duty which will give some pleasure to these girls is the cutting and arranging of flowers in the home. Some girls are not fond of reading or doing fancy work of their own: these will no doubt be glad to do a little plain sewing for the matron.

Tea at 5 o'clock would be the sociable meal of the day. With rare exceptions most of the patients in a convalescent home will be able to get up in the afternoon, and so the girls may be all together for this meal, which should be prepared for them, as it will add much to its enjoyment if they themselves have taken no part in its preparation. Matron should take her tea with them, and encourage the girls to chat freely with her, drawing them out to tell of the day's doings. They will not treat her with any less respect, but feel that they have in her a real and sympathetic friend. For tea they may have bread and butter, jam, salads, or fruit in season and home-made cakes. This meal should be taken out of doors in fit weather, otherwise in the sitting-room rather than the usual dining-room. The girls may wash up the tea-things and set the tables for the evening meal, which may consist of hot milk and biscuits, some evenings varied with soup, and this last meal should be served at 8 o'clock, the girls all being in bed by 9 o'clock.

The sitting-room should contain a piano, plenty of books, and indoor games for wet days.

### Decorative Materials.

DECORATIVE materials especially suitable for hospitals and institutions can be obtained from Jenson & Nicholson, Ltd., Goswell Works, Stratford, E. 15. A special feature is their "Acquatinta" washable distemper; this is a good permanent distemper, has an agreeable odour (a by no means negligible factor), and is produced in a variety of artistic shades. "Robbialac," an enamel presenting a compact and highly finished surface is especially suitable for theatre work. Among other preparations this firm supplies some good varnish stains.

